# Cholinesterase Inhibitory Activity of Alkylated Quinobenzothiazinium Salts

**DOI:** 10.3390/molecules31081346

**Published:** 2026-04-19

**Authors:** Sarka Stepankova, Andrzej Bak, Malgorzata Latocha, Violetta Kozik, Agata Kawulok, Josef Jampilek, Andrzej Zieba

**Affiliations:** 1 Department of Biological and Biochemical Sciences, Faculty of Chemical Technology, University of Pardubice, Studentska 573, 532 10 Pardubice, Czech Republic; 2 Institute of Chemistry, University of Silesia, Szkolna 9, 40-007 Katowice, Poland; andrzej.bak@us.edu.pl (A.B.); violetta.kozik@us.edu.pl (V.K.); agata.kawulok@gliwice.nio.gov.pl (A.K.); josef.jampilek@gmail.com (J.J.); 3 Department of Cell Biology, Faculty of Pharmaceutical Sciences, Sosnowiec Medical University of Silesia, Jednosci 9, 41-200 Sosnowiec, Poland; mlatocha@sum.edu.pl; 4 Department of Bone Marrow Transplantation and Oncohematology, Maria Sklodowska-Curie National Research Institute of Oncology, Gliwice Branch, Wybrzeze Armii Krajowej 15, 44-101 Gliwice, Poland; 5 Department of Chemical Biology, Faculty of Science, Palacky University Olomouc, Slechtitelu 27, 779 00 Olomouc, Czech Republic; 6 Department of Organic Chemistry, Faculty of Pharmaceutical Sciences, Sosnowiec Medical University of Silesia, Jagiellonska 4, 41-200 Sosnowiec, Poland

**Keywords:** ADME, Alzheimer’s disease, cholinesterase inhibitory activity, molecular docking, quinobenzothiazinium salts, similarity–activity landscape index

## Abstract

Ten substituted quinobenzothiazinium salts were tested for their ability to inhibit acetylcholinesterase (AChE) and butyrylcholinesterase (BChE). All the compounds inhibited AChE in the IC_50_ range of 0.03–0.658 µM, with 5,8,10-trimethyl-12*H*-quinolino[3,4-*b*][1,4]benzothiazin-5-ium chloride (**3d**) being the most potent inhibitor, with an IC_50_ value significantly better than that of the clinically used rivastigmine and galantamine and comparable to that of tacrine and donepezil. The IC_50_ values for BChE inhibition ranged from 0.34 to 4.25 µM; 5,9-dimethyl-12*H*-quinolino[3,4-*b*][1,4]benzothiazin-5-ium chloride (**3b**) exhibited the strongest BChE inhibitory activity and in general, all the investigated compounds were more potent inhibitors than rivastigmine and galantamine. Based on the calculated selectivity index values, they are rather preferential inhibitors of AChE. Cytotoxicity tests performed on normal human dermal fibroblasts (HFF-1) did not demonstrate any significant cytotoxicity under the tested conditions. The distance-oriented structure distribution for the studied molecules was related with the activity data using principal component analysis and hierarchical clustering analysis. (SAR)-based evaluation is reported to predict activity cliffs using a similarity–activity landscape index for the AChE inhibitory response values. Moreover, direct protein-mediated in silico methods were utilized to identify factors that may be relevant for quantitative (Q)SAR modeling. In practice, target-oriented molecular docking was used to organize the spatial distribution of the ligand property space for the anti-AChE system. In general, this series of alkylated quinobenzothiazinium salts with potent inhibitory activity against cholinesterases fulfills Lipinski’s rule of five based on in silico predictions and is also expected to have high absorption in the human gastrointestinal tract. All active derivatives are also expected to penetrate the blood–brain barrier, making them promising compounds for further research and possible use in Alzheimer’s disease therapy.

## 1. Introduction

Phenothiazines are a heterocyclic system composed of two benzene rings connected by a 1,4-thiazine ring. Compounds containing this structural fragment exhibit a variety of pharmacological properties [1,2,3]. The phenothiazine system with an alkylaminoalkyl chain at the thiazine nitrogen atom plays a key role in medicinal chemistry. Alkylaminoalkyl derivatives of phenothiazine were the structural basis of first-generation neuroleptics [4,5,6]. Compounds with this structure have also been used as antiemetics, antihistamines, antipruritics, analgesics, and anthelmintics [7,8,9]. One of the directions in the design and production of new drugs is based on modifications of basic structural fragments of known and already used drugs. Phenothiazines are an example of how small structural changes can affect the direction of pharmacological activity [10,11]. For several decades, considerable attention has been paid to the synthesis of new phenothiazine derivatives and the study of their biological activities. In this way, the phenothiazine system has become a platform for the design and synthesis of biologically active compounds. Recent reports include, among others, anticancer, antibacterial, antiviral, anti-inflammatory, and antioxidant activity, reversal of multidrug resistance, and potential treatment of Alzheimer’s and Creutzfeldt–Jakob disease [12,13,14,15,16,17,18,19,20,21].

Cholinesterase inhibitors (ChEIs) are of great importance in the treatment of various pathologies such as neurodegenerative diseases, glaucoma, myasthenia gravis, and gastrointestinal and urinary disorders [2,4]. In addition, they also serve as antidotes [22]. These drugs inhibit the enzymatic activity of acetylcholinesterase (AChE) and butyrylcholinesterase (BChE), enzymes that break down the neurotransmitter acetylcholine (ACh) [23]. Of particular importance is the use of ChEIs in the treatment of Alzheimer’s disease (AD). AD is the most common dementia, affecting more than 55 million people, and the fourth leading cause of death among the elderly worldwide. According to the World Health Organization, the number of people living with dementia across the world can be expected to increase to 152 million by 2050 [24]. AD is an age-related, multifactorial, neurodegenerative disease characterized by a progressive and irreversible decline in cognitive function. The pathological process is associated with the selective loss of neurons and synapses, caused by the deposition of amyloid plaques, which leads to disruption of synaptic communication between neurons. The disease is clinically manifested by progressive impairment of memory, learning, language, orientation, decision-making and judgment. Oxidative stress and neuroinflammation also play a significant role in the progression of the disease [25,26,27]. Currently, three groups of drugs are approved and used for the treatment of AD, which differ in their mechanism of action. These are (1) cholinesterase inhibitors (rivastigmine, galantamine and donepezil), (2) *N*-methyl-d-aspartate receptor antagonist (memantine), and (3) humanized monoclonal antibodies (lecanemab and donanemab). Memantine can be administered alone or, in indicated cases, in combination with ChEIs [28,29].

(Aza)phenothiazine derivatives have been recognized as interesting/promising molecules interacting with both AChE and BChE [30,31,32,33,34,35]. In general, however, there are not many reports on the influence of the location of substituents/groups within the phenothiazine or azaphenothiazine system on the interaction of these compounds. The influence of different types of pharmacophoric groups on the direction of biological interaction has been extensively analyzed by our group. In previous works, we have described the synthesis of tetracyclic azaphenothiazine derivatives [36]. The developed synthetic method allows for structural modification of the phenothiazine system by introducing different pharmacophoric groups into different positions of the tetracyclic quinobenzothiazine system. The synthesized compounds showed interesting anticancer and antimicrobial properties.

Systematic observations of trends in chemical structure modifications that result in corresponding changes in biological responses for a congeneric series of molecules are essential for multidimensional quantitative structure–activity relationship (mD-QSAR) studies [37]. Transitioning from intricate biological relationships to straightforward QSAR models requires the quantitative mapping of experimental properties or calculated descriptors into ADME (absorption, distribution, metabolism, and excretion)-driven molecular potency. That can support the synthetic efforts at the decision-making phases of hit → lead → seed → drug design [38]. Despite certain limitations, the examination of distance-related similarities in structurally similar compounds is a standard procedure that significantly aids in both quantitative and qualitative SAR mapping. The concept of defining a numerical measure of intermolecular similarity is commonly utilized in chemical information systems [39]. A collection of characteristics may be represented by a bit-string format to compute a quantitative measure of structural similarity between objects, such as the Tanimoto coefficient. A perplexing element of many SAR analyses is the occurrence of the activity hotspots or an activity cliff within the structure–activity landscape [40]. Identifying the activity hotspots offers valuable insights into a central question in medicinal chemistry regarding how molecular rearrangement influences the activity profile. Activity cliffs are often quantified numerically through various fingerprint representations or similarity metrics; for example, the structure–activity landscape index (SALI) has been suggested to assess the smoothness of SAR [41].

In target-guided QSAR procedures, the complementary (bio)effector binding mode is obtained based on the intrinsic relationship between the atomic coordinates of both the receptor and ligand in the binding/active site [42]. The chosen spatial distribution of the ligand property space is influenced by the corresponding mapping of the target’s steric, electronic, or lipophilic characteristics. The effective site-directed QSAR method known as docking can be applied when the geometry of the macromolecule, or at least reliable homology models, are available. Molecular docking is a widely used technique in structure-based drug design (SBDD). However, this approach does not consistently yield quantitative correlations between in silico calculations and actual activity assays [43].

The paper focuses on investigating whether and how our alkylated quinobenzothiazinium salts affect cholinesterases. Advanced virtual screening and ADME profiling techniques, which ensure robust selection of candidates with promising drug-like properties, were used for the ADME characterization of our compounds. Following the common practice, the intermolecular similarity of the investigated derivatives was estimated in the multidimensional space (mDS) using principal component analysis (PCA) and hierarchical clustering analysis (HCA). Moreover, the distance-oriented property distribution for those compounds was correlated with the activity data. Finally, planar (2D) and spatial (3D) maps of the host–target interactions were created for the most potent compound and subsequently compared with the marketed drug molecule (tacrine) using the docking methodology.

The findings of the study could thus significantly contribute to the development of novel therapeutic agents for AD and offer insights into the structure–activity relationships of alkylated quinobenzothiazinium salts and their potential pharmacological applications.

## 2. Results and Discussion

### 2.1. Chemistry

**Scheme 1:**
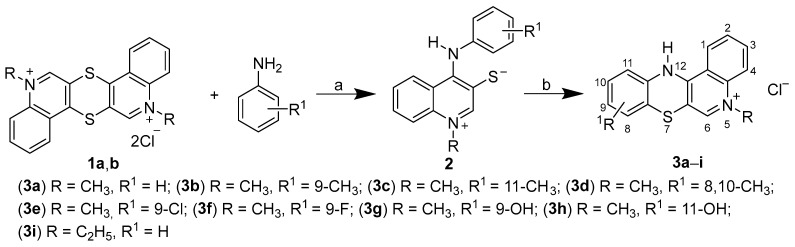
Synthesis of 5-alkyl-12(*H*)-quino[3,4-*b*][1,4]benzothiazinium salts **3a**–**i**. Reagents and conditions: (a) Py, room temp.; (b) Py 80 °C, O_2_, and HCl.

In previous work, a unique, original method for the synthesis of azaphenothiazine derivatives was presented [36], which enables the formation of derivatives containing specific substituents at different positions of the tetracyclic quinobenzothiazinium system. This method involves the reaction of thioquinanthrenediinium bis-chloride (**1**) with substituted isomeric anilines. The intermediate product of these reactions is a betaine system with the structure of 1-methyl-4-(phenylamino)quinoline-3-thiolane (**2**), the cyclization of which leads to the thiazine ring and the formation of 5-alkyl-12(*H*)-quino[3,4-*b*][1,4]benzothiazinium salts **3**. Control of the cyclization reaction parameters allows for the selective course of the reaction and the introduction of various types of substituents at positions 9, 10, and 11 of the quino[3,4-*b*][1,4]benzothiazine scaffold (see Scheme 1). The structures of most of the compounds tested in this work have been described in previous work [14]. 5-Methyl-8,10-dimethyl-12(*H*)-quino[3,4-*b*][1,4]benzothiazinium chloride (**3d**) containing two methyl groups in positions 8 and 10 of the tetracyclic quinobenzothiazinium system was obtained by reaction of thioquinanthrenium salt **1** with 3,5-dimethylaniline. The structure of the obtained compound was confirmed by ^1^H, ^13^C NMR and HRMS methods.

**Scheme 2:**
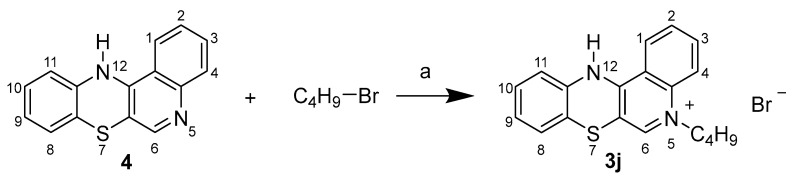
Synthesis of 5-butyl-12(*H*)-quino[3,4-*b*][1,4]benzothiazinium chloride **3j** by alkylation of 12(*H*)-quino[3,4-*b*][1,4]benzothiazine (**4**). Reagents and conditions: (a) reflux.

Compound **3j** containing butyl at the quinoline nitrogen atom was obtained in the alkylation reaction of quino[3,4-b][1,4]benzothiazine **4** with butyl bromides [15,36] (see Scheme 2). The structures of all compounds are shown in Table 1.

### 2.2. Cytotoxicity

First, all compounds were subjected to cytotoxicity testing on normal human dermal fibroblasts (HFF-1). Compounds are considered to have a favorable safety profile if they exhibit cytotoxicity expressed as IC_50_ > 30 µM on normal, non-cancerous fibroblast lines [44,45]. As can be seen from the values (IC_50_) shown in Table 1, the IC_50_ values of compound **3i** and compound **3b** are 30.8 ± 1.6 µM and 35.8 ± 1.2 µM, respectively. The starting compound **3a** has an IC_50_ > 40 µM, with the values of the other derivatives >100 µM. Considering the values of cholinesterase inhibition, it can be stated that no significant cytotoxicity was observed under the tested conditions, indicating that these compounds are suitable for subsequent biological studies.

### 2.3. Inhibition of Cholinesterases

The inhibitory activity of the investigated derivatives against electric eel AChE and horse serum BChE was studied using the modified Ellman’s method, which is a standard approach for in vitro screening of potential cholinesterase inhibitors. The ability of the compounds to inhibit these enzymes was expressed as the IC_50_ value representing the concentration of inhibitor that causes a 50% reduction in enzyme activity. Selectivity indexes (SIs) were also calculated: SI for AChE as the ratio of IC_50_ for BChE/AChE, and SI* for BChE as the ratio of IC_50_ for AChE/BChE. Rivastigmine, galantamine, tacrine and donepezil were used as standards for comparison. From the values in Table 1 it is evident that all compounds inhibit AChE in the IC_50_ range from 0.03 µM (compound **3d**) to 0.658 µM (compound **3i**), with derivative **3d** being the most potent inhibitor, followed by **3e**, with IC_50_ values that are significantly better than those of the clinically used rivastigmine and galantamine and comparable to those of tacrine and donepezil. The IC_50_ values for BChE inhibition range from 0.34 µM (compound **3b**) to 4.25 µM (compound **3g**). However, these derivatives are also more potent inhibitors than rivastigmine and galantamine. From the calculated values of the selectivity indices, it is clear that these are rather preferential inhibitors of AChE, especially compounds **3d** and **3e**, whose SIs for AChE are 66 and 15.

In terms of AChE inhibition, it can be stated that replacing methyl with ethyl on the heterocyclic nitrogen (compounds **3a**/**3i**) leads to a decrease in activity. It is noteworthy that replacing methyl with butyl gives a derivative with slightly improved activity (compare **3a**/**3j**). Therefore, it can be stated that the basic unsubstituted methylated scaffold of compound **3a** is not optimal, but disubstitution at positions 8 and 10 with a rather lipophilic, electroneutral substituent seems to be advantageous (compound **3d**), followed by monosubstitution at position 9 with a lipophilic and rather electron-withdrawing substituent. On the other hand, using a polar hydroxyl group or moving the substitution to position 11 reduces the AChE inhibitory activity.

In terms of a substitution positively affecting BChE inhibitory activity, the replacement of the methyl group on the heterocyclic nitrogen with ethyl and butyl is disadvantageous. On the contrary, in comparison with AChE, in addition to the basic skeleton **3a**, a smaller, rather electroneutral and more lipophilic substituent at position 9 (compounds **3b** and **3f**) appears to be an advantageous substitution. Transfer of the polar substituent (OH) to position 11 (compound **3h**) probably changes the binding disposition in the enzyme cavity and optimizes binding (compare **3g**/**3h**). Disubstitution, or substitution with a non-hydrophilic methyl at position 11, is disadvantageous compared to binding to the AChE cavity.

**Figure 1:**
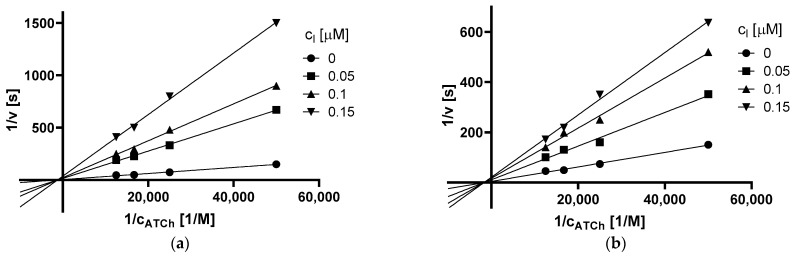
Lineweaver–Burk plot for derivatives **3d** (**a**) and **3e** (**b**) inhibiting AChE non-competitively. (The measurements were done in duplicates, and the points are expressed as mean values.) (ATCh—acetylthiocholine).

**Figure 2:**
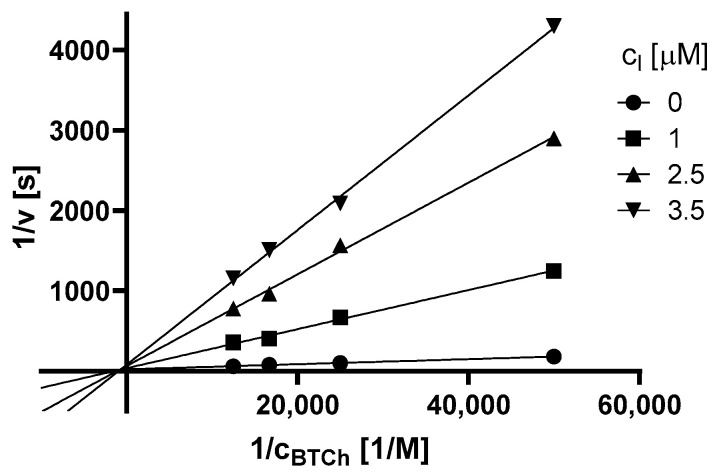
Lineweaver–Burk plot for the derivative **3b** inhibiting BChE non-competitively. (The measurements were done in duplicates, and the points are expressed as mean values.) (BTCh—butyrylthiocholine).

The type of inhibition of the most active inhibitors of AChE (compounds **3d** and **3e**) and BChE (compound **3b**) was also determined. The type of inhibition can be identified using a Lineweaver–Burk plot [46] and by comparing the kinetic parameters of the Michaelis constant (K_M_) and the maximum velocity (V_m_) determined for the uninhibited and inhibited reaction. The nature of the changes in the K_M_ and V_m_ values, together with the intersection of the lines in the Lineweaver–Burk plot, distinguishes the type of inhibition. Reversible enzyme inhibitors are classified as competitive, classical non-competitive, non-competitive, and mixed (also referred to as mixed non-competitive inhibition). The results are illustrated in Figure 1 and Figure 2. From the obtained results it is evident that all the compounds act as classical non-competitive inhibitors of both AChE (compounds **3d** and **3e**) and BChE (compounds **3b**).

### 2.4. In Silico ADME Profiling

The optimal cholinesterase inhibitor should not only exhibit significant enzyme inhibition, but also low cytotoxicity to mammalian cells and suitable physicochemical properties for crossing the blood–brain barrier (BBB) and drug-likeness. The investigated compounds and standards were in silico-evaluated for their physicochemical properties and whether they satisfy Lipinski’s rule of five. Table 2 summarizes molecular weights, log *P* values, number of H-donors, number of H-acceptors, and tPSA (topological polar surface area) values. It is evident from Table 2 that all investigated compounds satisfy Lipinski’s rule of five with no violation and demonstrate a high oral bioavailability score of 0.55. The tPSA values of all investigated compounds indicate good membrane permeability and the possibility of crossing the BBB [47,48,49].

**Figure 3:**
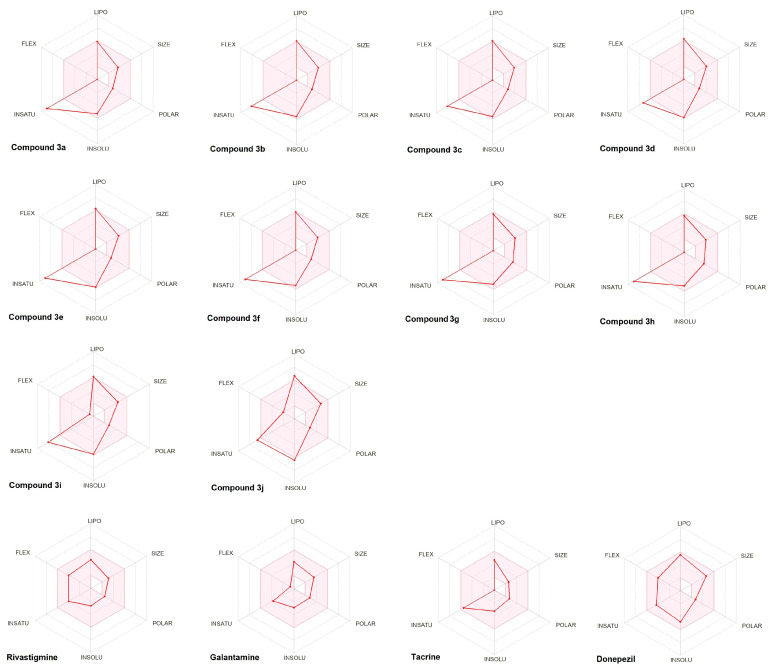
Bioavailability radar plots of compounds **3a**–**j** and standards.

A bioavailability radar is displayed for quick assessment of drug similarity (see Figure 3). The radar representation illustrates six key physicochemical parameters influencing oral bioavailability, including lipophilicity, size, polarity, solubility, saturation, and flexibility. Each property is defined by a descriptor of SwissADME and a range of optimal values is depicted as a pink area. To be estimated as drug-like, the red line of the compound under study must be fully included in the pink area. Any deviation represents a suboptimal physicochemical property for oral bioavailability. Figure 3 shows that for all new compounds investigated, the saturation parameter (representing the ratio of sp^3^-hybridized carbons over the total carbon count of the molecule) deviates from the optimal physicochemical space.

**Figure 4:**
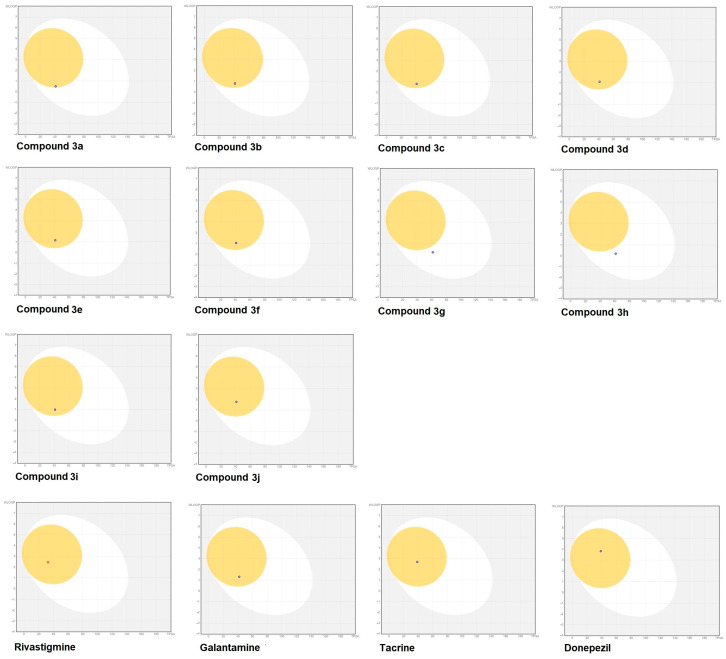
The BOILED-Egg plots of compounds **3a**–**j** and standards.

The BOILED-Egg model [50], an intuitive graphical classification model, includes predictions of two key ADME parameters, i.e., passive absorption in the human gastrointestinal tract (GIT) and permeation through the BBB (Figure 4). This classification model relies on only two physicochemical descriptors (WLOGP and tPSA for lipophilicity and apparent polarity). The yolk represents a physicochemical space for highly probable BBB permeation, and the white represents a physicochemical space for highly probable passive absorption in the human GIT. As can be seen from Figure 4, only compounds **3g** and **3h** are predicted to be well-absorbed but not accessing the brain (in the white). For all other compounds investigated, both passive absorption in the GIT and passive crossing of the blood–brain barrier are predicted (in the yolk covering the area of the white).

### 2.5. Modeling SAR and Docking

#### 2.5.1. Similarity Evaluation of Descriptor-Derived Space

**Figure 5:**
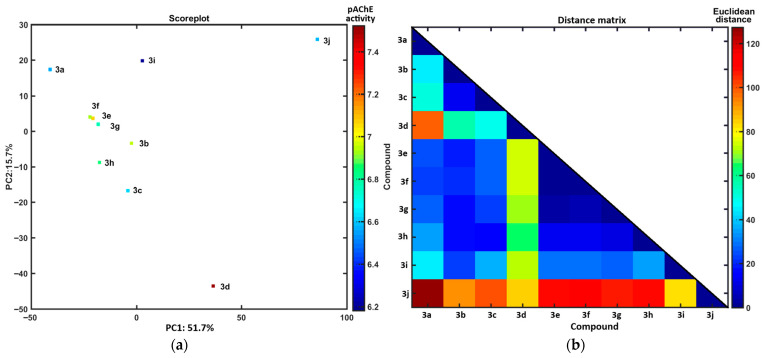
Projection of molecules **3a**–**3j** on the plane defined by the first versus second PC for Dragon descriptors with AChE activity in the logarithmic scale (**a**); color-coded matrix of the Euclidean distances calculated using PC1 and PC2 vector (**b**).

The assessment of molecular similarity is widely applied in ligand-based studies [51]. The tendency of the investigated compounds to cluster can be analyzed by examining their pairwise (dis)similarities in the multidimensional (mD) descriptor space generated in silico with Dragon 6.0 software [52]. To graphically analyze similarity in the mD molecular space, where each compound is characterized by a set of 2580 descriptors, the PCA method can be applied to the resulting matrix X_10×2580_. Each row in the matrix X represents an individual molecule (object) and the columns depict parameters (variables) [53]. Overall, PCA is a projection technique used to model multivariate datasets with a relatively small number of principal components (PCs). It helps in the reduction in the dimensionality of the data (mD → 2/3D), thereby enabling easier visualization and interpretation. In practice, the input matrix X is decomposed into two orthogonal matrices: scores (T) and loadings (P). The PCs are formed as a linear combination of the original variables calculated to capture the maximum variance present in the dataset. The appropriate number of relevant PCs_(f)_ is determined based on the cumulative percentage of variance explained by successive PCs. Using the obtained score vectors, similarity between compounds can also be assessed through distance-based measures, for instance the Euclidean distance. During the pre-processing stage, the X_10×2580_ matrix was centered and standardized to equalize the variance of individual descriptors. Considering the percentage of the modeled data variance, a limited number of key PCs is identified; the effectiveness of PCA-driven data compression strongly depends on the number of resulting uncorrelated variables. In this study, the first three PCs explain approximately 79.6% of the total variance in the dataset, whereas the first two PCs account for about 67.4%. Consequently, the analyzed molecules were mapped onto the plane defined by PC1 and PC2 and additionally color-coded according to the AChE activity in the logarithmic scale, as shown in Figure 5.

Noticeably, the examined molecules (**3a**–**3j**) are generally classified into three structurally related groups according to the first principal component (PC1), where the first one (PC1 ≤ 0) contains the majority of compounds. The second cluster (0 < PC1 < 50) comprises the most active AChE molecule **3d** bearing two -CH_3_ groups at position 8 and 10 of the benzene ring, while the third one (PC1 > 75) contains the structurally incoherent molecule **3j** with butyl linked to the heterocyclic nitrogen. As expected, the unsubstituted compound **3a**, doubly substituted molecule **3d** and butyl-containing molecule **3j** exhibit extreme values for both PC1 and PC2, which places them at the outer boundaries of the PC1–PC2 projection plane. The Euclidean distances (d) were computed for all molecules, based on the PC1 and PC2 coordinates for all molecules, and subsequently presented in the form of a color-coded triangular matrix, as depicted in Figure 5b. The distance-based similarity assessment further confirms the presence of three distinct clusters of compounds, characterized by low (molecules **3e**–**3i**), moderate (molecule **3d**), and high (molecule **3j**) Euclidean distances (see Figure 5b).

**Figure 6:**
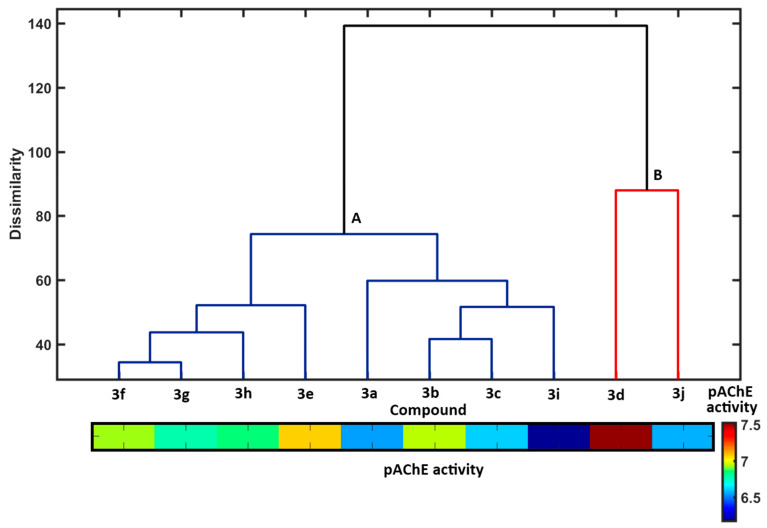
HCA dendrogram of molecules **3a**–**3j**.

In general terms, hierarchical clustering analysis (HCA) generates a grouping pattern of objects (molecules), which can be visualized as a two-dimensional dendrogram based on Euclidean distance. In a dendrogram-based representation, the *X*-axis indicates the order of the objects (or variables), whereas the *Y*-axis reflects the level of similarity or dissimilarity between them [54]. The similarity-driven classification of compounds largely depends on the linkage criterion applied to define clusters. For this purpose, the Ward linkage method is most commonly used. Overall, the clustering pattern noted in the previous PCA is corroborated by the HCA method, as illustrated in Figure 6. Consistent with our earlier PCA results (see Figure 5a), molecules **3a**–**3j** group into two main clusters: A and B. Cluster B contains only two molecules, characterized by the extreme values of the AChE activity (compounds **3d** and **3j**), and therefore cannot be further divided into two additional subgroups.

**Figure 7:**
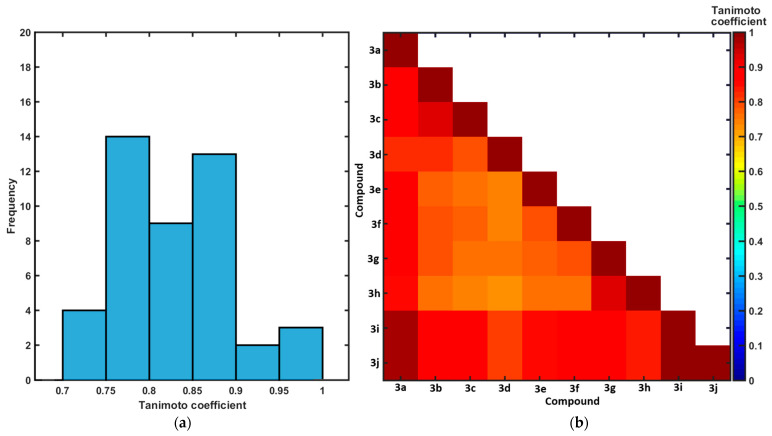
Distribution of Tanimoto coefficients (**a**); the triangular matrix of Tanimoto coefficients (**b**) for molecules **3a**–**3j**.

The Tanimoto coefficient (Tc) is favored as a quantitative metric for describing the pairwise relatedness among molecules in the descriptor-based chemical space (CS). Thus, the structural similarity of molecules **3a**–**3j** was also measured between pairs of OpenBabel fingerprints using the Tanimoto coefficient [55]. The graphical representation of Tanimoto coefficients for the examined set of compounds is depicted in Figure 7a, where the highest frequency is noted in the range of 0.9 ≤ Tc ≤ 0.8. Additionally, a symmetrical matrix of Tc_10×10_, which displays the numeric values of Tanimoto coefficients for all possible combinations of molecules **3a**–**3j**, was computed and presented as a triangular matrix in Figure 7b. The highly potent AChE compound **3d** demonstrated a structural similarity (Tc ≥ 0.8) to molecules which include at least two carbon atoms attached to the common scaffold, while molecules with a single –CH_3_ group (**3e**–**3h**), bonded to the heterocyclic nitrogen ring, are characterized by lower values of T_C_. It is not unexpected that the unsubstituted compound **3a**, serving as a type of scaffold, shares a structural similarity with all the other compounds.

**Figure 8:**
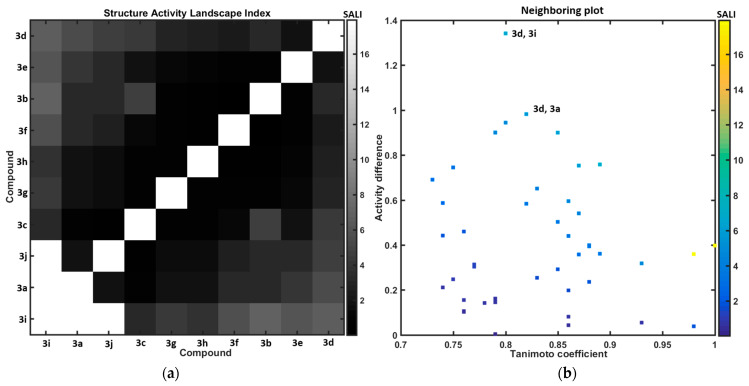
The gray-scaled structure activity landscape index (SALI) plot with molecules ordered according to increasing pAChE activity (**a**); the neighboring plot (**b**) for molecules **3a**–**3j**.

The systematic profiling of structure–activity indexes (SALI) involves the pairwise comparison of chemical chemotypes and their corresponding biological responses. It aims to create a visualization framework for understanding SAR trends (including continuity regions and activity cliffs). The graphical representation of activity cliffs heavily relies on the availability of chemically similar molecules (such as stereoisomers) that exhibit significant variations in activity (e.g., three orders of magnitude). Clearly, SALI approaches infinity for structurally related compounds (with a similarity index Tc ≈ 1), which show considerable differences in biological response values. Consequently, these values are usually substituted with the highest SALI parameters. The symmetrical heatmap illustrating the SALI characteristics for the examined molecules **3a**–**3j** is displayed in Figure 8a, with axes arranged according to the increased values of the AChE activity (expressed in the logarithmic scale) and a legend indicating the SALI values. Generally, two categories of SALI terminal spots can be identified, with the white spots indicating the molecules that display the highest SALI values. The brighter blocks positioned in the lower right corner of the map (or, symmetrically, in the upper left corner) denote pairs of compounds, where even minor structural changes can enhance or entirely eliminate the biological activity (magic methyl paradox). Not surprisingly, the most potent molecule **3d** with double –CH_3_ groups in position 8 and 10 of the benzene ring can potentially form activity cliffs with inactive molecule **3i** that have one ethyl substituent attached to the heterocyclic nitrogen (see Figure 8a). To identify the unfavorable structural changes, a neighborhood plot was created, where structurally similar molecules are represented based on their activity differences and are color-coded according to their SALI values, as illustrated in Figure 8b. Active molecule **3d** is positioned alongside inactive molecules in the rough/rocky section of the neighborhood plot (Tc > 0.80 and ∆log(AChE) > 1.0), indicating areas for further dense sampling of the specified SAR variations.

#### 2.5.2. Molecular Docking Study

**Figure 9:**
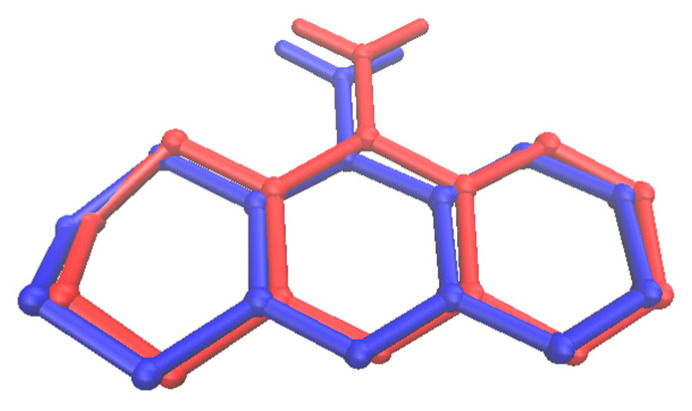
Docking-based alignment of PubChem-retrieved tacrine (blue color) on crystal geometry of tacrine (red color).

To gain a more comprehensive understanding of guest–host interactions, 3D ligand-based methods can be integrated with site-directed protein docking techniques. This may be particularly helpful when a spatial geometry or homology model of the target that influences the ADME profile is available [56]. In docking-driven structure–activity relationship (SAR) modeling, the enthalpically and/or entropically favorable factors between the ligand and target can be inferred from the protein (or enzyme) binding site as well as from the properties or descriptors of the ligand-coded structural space. Although establishing precise relations between ligand–target interactions and pharmacological or toxicological outcomes remains uncertain, the effectiveness of intuitive docking techniques in generating guest-bound configurations within structure-based drug design (SBDD) is broadly acknowledged. To efficiently compare findings from SBDD for AChE activity, it is imperative to obtain crystallographic data involving commercially available drug molecules, characterized by the comparable values of AChE response, e.g., tacrine (1,2,3,4-tetrahydroacridin-9-amine). Hence, the crystallographic structure of AChE, determined at a resolution of 2.85 Å in its liganded form (holo) with tacrine (THA), was retrieved from the Protein Data Bank (PDB entry code: 7XN1) [57]. The AutoDock Vina 1.2 program was employed to (re)dock the tacrine (downloaded from the PubChem database, CID 1935) into the active site of the enzymatic chain A (with the crystal water retained) with the grid box of 15 × 15 × 15 Å, centered on the THA amine group [58]. In fact, energetically favorable pose 1 (the conformation and relative orientation) of the PubChem-derived THA molecule fits well to the crystal geometry of tacrine (see Figure 9). That is because the implementation of a new scoring function in AutoDock Vina greatly improved the speed and accuracy of the docking algorithm.

**Figure 10:**
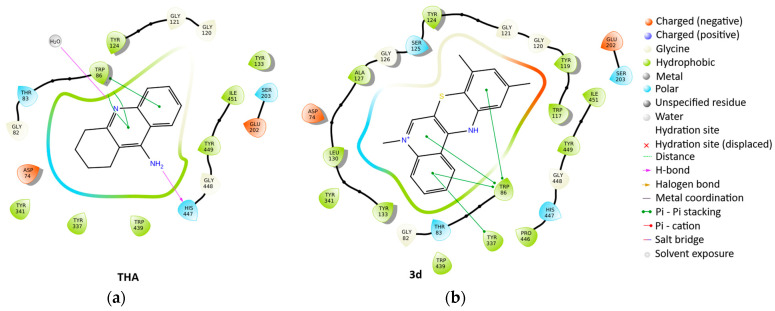
2D–based comparison of binding pattern for THA (**a**), and the most active AChE inhibitor **3d** (**b**), produced by the Maestro program.

**Figure 11:**
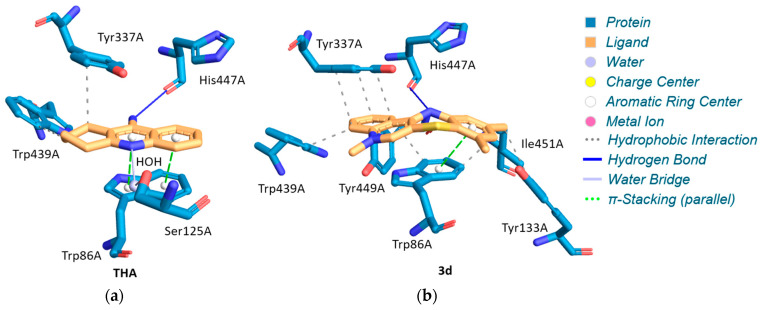
3D-based comparison of binding pattern for THA (**a**), and the most active AChE inhibitor **3d** (**b**), produced by the PLIP software.

Consequently, the entire anti-AChE population was validated by an automated docking procedure in order to compare the interaction patterns between the marketed drug (tacrine) and the examined set of molecules **3a**–**3j**. Despite its structural variations from the reference THA drug, special emphasis was put on the most potent AChE inhibitor **3d**, due to its high AChE activity (comparable with tacrine). The depiction of the interactions between host and guest was made using both planar (2D) and spatial (3D) maps created by the Schrödinger Maestro 12.3 software and the Protein-Ligand Interaction Profiler (PLIP) [59]. It revealed three dominant types of interacting modes: hydrophobic, hydrogen-bonding and π-stacking ones. The binding 2D/3D patterns for the commercial drug THA, as well as the most effective anti-AChE molecule **3d**, are illustrated in Figure 10 and Figure 11, respectively.

In fact, an automated docking procedure positioned the energetically preferable pose 1 of tacrine, similarly to the previously reported structures [57]. That favors the hydrophobic and π-stacking interactions, where THA and molecule **3d** are being sandwiched between Trp86 and Tyr337, as presented in Figure 11. Contrary to tacrine, the multiplicity of hydrophobic interactions was foreseen by the docking protocol for the most active molecule **3d**, including the following set of amino acid residues: Trp86, Tyr133, Tyr337, Trp439, Tyr449 and Ile451. The amine moiety of tacrine and the non-aromatic nitrogen of compound **3d** seem to play a vital role as hydrogen bond donors, while forming the hydrogen bond with the carbonyl oxygen of His447. Since crystal water molecules may have an imperative role in mediating ligand recognition, the water molecules of the binding cavity were retained. That in turn resulted in the formation of a bridged hydrogen bond between the ring nitrogen of tacrine (N7 atom), crystal water 709 and Ser125 (see Figure 10a); however, no such bonding pattern was observed for the most active molecule **3d** (see Figure 10b). The binding affinities of energetically favorable pose 1 for the analyzed set of molecules **3a**–**3j** and marketed drugs, approximated using the AutoDock Vina scoring function, are presented in the Supplementary Materials (Table S1). In general, the relationship between binding affinity and biological activity is not linear; however, the calculated correlation coefficient for the examined molecules **3a**–**3j** indicates a pretty strong correlation (r = 0.74).

## 3. Materials and Methods

### 3.1. Chemistry

Melting points are uncorrected. NMR spectra were recorded using a Bruker Ascend 600 spectrometer (Bruker, Billerica, MA, USA). To assign the structures, the following 2D experiments were employed: ^1^H/^13^C gradient-selected HSQC and HMBC sequences. Standard experimental conditions and standard Bruker programs were used. The ^1^H NMR and ^13^C NMR spectral data are provided relative to the TMS signal at 0.0 ppm. HR mass spectra were recorded with a Bruker Impact II (Bruker, Billerica, MA, USA).

#### Synthesis of 5,8,10-Trimethyl-12H-quinolino[3,4-b][1,4]benzothiazin-5-ium Chloride (**3d**)

3,5-dimethylaniline (0.121 g, 2.5 mmol) was added to a mixture of bis-chloride (**1**) (0.419 g, 1 mmol) in 10 mL of dry pyridine and the whole mixture was mixed at 80 °C for 12 h. The mixture was cooled down to room temp., and the formed precipitate was filtered off and washed with ether. The raw product was purified through recrystallization from ethanol.

Yield: 92%; m.p. = 158–159 °C; ^1^H NMR (DMSO, 600 MHz)—δ (ppm): 2.05 (s, 3H, CH_3_), 2.19 (s, 3H, CH_3_), 3.45 (s, 3H, CH_3_), 6.28–6.33 (m, 1H, H_arom_), 6.57–6.61 (m, 1H, H_arom_), 6.96–6.99 (m, 1H, H_arom_), 7.17–7.24 (m, 2H, H_arom_), 7.46–7.52 (m, 1H, H_arom_), and 8.15–8.20 (m, 1H, H_arom_); ^13^C NMR (DMSO, 150.9 MHz)—δ (ppm): 18.17 (CH_3_), 20.60 (CH_3_), 39.43 (NCH_3_), 103.43 (C), 115.68 (C), 120.00 (C), 121.97 (C), 123.80 (C), 123.84 (C), 124.94 (C), 130,09 (C), 131.73 (C), 132.33 (C), 133.94 (C), 134.44 (C), 140.25 (C), 140.91 (C), and 151.67 (C); ESI-HRMS Calcd. for C_18_H_17_N_2_S ([M]^+^) = 293.1112, found = 293.1116.

### 3.2. Biological Evaluation

#### 3.2.1. Cytotoxicity Assay

Cell lines: This study examined normal skin fibroblasts obtained from the foreskin of a newborn (HFF-1 line, ATCC). Cultures were conducted under the following conditions: 5% CO_2_, 37 °C, and constant humidity. DMEM medium with 10% fetal bovine serum and a penicillin/streptomycin antibiotic mixture were used. Cells were seeded into 96-well plates (5000 cells/well) and incubated for 24 h. After this time, the medium was replaced with new medium containing the tested derivatives. The derivatives were dissolved in DMSO, and the appropriate concentrations of the tested compounds were then prepared in the medium. After 72 h of incubation, HFF-1 cell viability was assessed using the WST-1 assay.

#### 3.2.2. WST-1 Test

The WST-1 (Roche Diagnostics, Mannheim, Germany) test was used to spectrophotometrically determine the relative number of cells based on their metabolic activity. It is used to study cell proliferation in response to various factors, such as growth factors, mitogens, and cytotoxic substances, including chemical compounds used in medicine. The method involves adding the WST-1 reagent, which contains tetrazolium salts with a delicate red color, to wells containing cells. Under the influence of cellular enzymes (including succinate dehydrogenase), the tetrazolium salts undergo a reduction reaction, resulting in the formation of a dark red formazan. A higher number of viable cells translates into a higher number of mitochondrial dehydrogenases, among others, and greater overall activity. The concentration of the produced dye is then determined spectrophotometrically by measuring absorbance at a wavelength of λ = 450 nm.

#### 3.2.3. Inhibitory Activity of Cholinesterase (AChE and BChE)

The modified Ellman’s method [60] was used to determine the inhibitory activity of the studied compounds against AChE and BChE. Acetylthiocholine (ATCh) was used as the substrate for AChE, while butyrylthiocholine (BTCh) was used for BChE. Uninhibited and inhibited substrate hydrolysis (ATCh/BTCh) catalyzed by AChE/BChE was carried out in phosphate-buffered saline (PBS, 0.1 M, pH 7.4) at room temperature. The enzyme activity in the final reaction mixture (2000 µL) was 0.2 U/mL, the concentration of ATCh/BTCh was 40 µM and the concentration of 5,5′-dithiobis(2-nitrobenzoic acid) (DTNB) was 100 µM. The investigated derivatives were dissolved in DMSO to a concentration of 0.01 M and diluted as needed with demineralized water (conductivity 3 µS; equipment supplier BKG Water Treatment, Hradec Kralove, Czech Republic). To determine the IC_50_ values of all studied compounds and standards (rivastigmine, galantamine, tacrine and donepezil), five different inhibitor concentrations were used in the final reaction mixture. All experiments were performed in triplicate. The time dependences of absorbance at a wavelength of 412 nm were monitored and the reaction rates for uninhibited (v_0_) and inhibited (v_i_) reactions were calculated. Subsequently, the dependence of v_0_/v_i_ on the inhibitor concentration was plotted and the IC_50_ values were calculated from the obtained regression curve equations for y = 2 (based on the definition of IC_50_).

The type of inhibition was also determined for the two most potent AChE inhibitors and the most potent BChE inhibitor using a Lineweaver–Burk plot [46]. The measurement procedure was similar to that for IC_50_ determination. Reactions were performed in PBS (0.1 M, pH 7.4) at room temperature. The AChE/BChE activity in the final reaction mixture was 0.2 U/mL, the concentration of ATCh/BTCh was 20–80 µM, and the concentration of DTNB was 100 µM. Again, the time dependences of absorbance at a wavelength of 412 nm were monitored in the presence and absence of inhibitor and the reaction rates were calculated. All experiments were performed in duplicate and the average values of reaction rates were used to construct the Lineweaver–Burk plot. From the obtained regression curve equations, the values of the Michaelis constant and the maximum velocity were calculated and the type of inhibition was determined.

### 3.3. ADME Profile

SwissADME [61] was used to evaluate the physicochemical properties of the studied compounds and standards. This is an effective web tool that allows one to compute physicochemical descriptors and also predict ADME parameters, pharmacokinetic properties, the medicinal nature and the suitability of small molecules for medicinal chemistry.

The relationship between pharmacokinetic and physicochemical parameters was delineated by the so-called Lipinski’s rule of five [62]. This is a rule of thumb used in drug discovery to predict the likelihood of a chemical compound having good oral bioavailability, based on four physicochemical properties. This rule predicts that poor absorption or permeation is more likely when there are more than five hydrogen-bond donors and 10 (5 × 2) hydrogen-bond acceptors, a molecular weight greater than 500 (5 × 100) and a calculated octanol/water partition coefficient (CLog*P*) greater than 5. Violating more than one of these guidelines reduces the likelihood of adequate absorption.

In addition to log *P*, topological polar surface area (tPSA) has been identified as another representative parameter for CNS-targeting drugs. It is a 2D medicinal chemistry descriptor representing the surface sum of polar atoms (mostly oxygen, nitrogen, and attached hydrogens) in a molecule. It correlates well with 3D polar surface area. TPSA is a critical, fast-calculated medicinal chemistry metric used to predict drug absorption, permeability, and bioavailability [63,64]. Generally, molecules with a PSA > 140 Å^2^ have poor cell membrane permeability. For molecules to effectively cross the BBB, a PSA of less than 70–90 Å^2^ is usually required [65,66].

To perform ADME profiling, structures of the investigated compounds (created in ChemDraw 21.0.0.) were uploaded as input to the SwissADME server. These were subsequently converted to SMILES format. The output panel is divided into different sections: chemical structure and bioavailability radar, physicochemical properties, lipophilicity, water solubility, pharmacokinetics, drug-likeness, and medicinal chemistry. In addition, it is possible to display a graphical output, a BOILED-Egg model, which predicts the ability of the investigated compound to be passively absorbed in the GIT (white region) and passively cross the BBB (yellow region). The obtained outputs were processed into Table 2 (fulfillment of Lipinski’s rule of five) and Figure 3 (bioavailability) and Figure 4 (passive absorption in human GIT and passive crossing the BBB).

### 3.4. Model Building

The CACTVS/csed (Xemistry GmbH, Königstein, Germany) and CORINA 4.4.0 (Molecular Networks GmbH, Leeds, UK) structural editors were utilized to create 3D molecular models. The conversion of data formats was performed using an OpenBabel interchange file format converter. The Sybyl-X 2.0 package (Certara, Radnor, PA, USA) installed on a DELL workstation operating with Debian 12 was used to conduct the molecular modeling simulations. To initially optimize the spatial geometry of the compound, the MAXMIN2 module incorporated in Sybyl-X was employed along with the standard Tripos force field (using the POWELL conjugate gradient algorithm) and a convergence criterion of 0.01 kcal/mol for the energy gradient. The electrostatic potential values were computed using the Gasteiger–Hückel method. A trial alignment consisting of 10 ordered atoms was generated for unsubstituted compound **1** with an FIT procedure to encompass the complete bonding topology in the maximal common structure (MCS).

### 3.5. Principal Component and Hierarchical Clustering Analysis

Mapping the molecular diversity within the vast chemical space (CS) into the corresponding biological or property space typically necessitates multi-dimensional descriptor representations. A particular molecule can be represented by a collection of structural (S) and physicochemical (P) properties arranged in a vector, which specifies an object within the CS. The molecular distribution of compounds generated empirically (FCS) and virtually (VCS) can be visually analyzed using a linear projection technique known as principal component analysis (PCA). PCA serves as a projection technique aimed at modeling multivariate data with a comparatively small number of so-called principal components. These principal components (PCs) are formulated as linear combinations of the original variables to maximize data variance description. The PCA model transforms the information found in a data matrix into principal components, specifically scores and loadings. The score matrix offers insights into the similarities between data objects, while the loading matrix enables examination of the similarities among variables and their contributions to the formation of specific principal components. A PCA model with *f* principal components for a data matrix *X* can be expressed as follows:

*X* = *TP^T^*+ *E* (1)

where *X* is a data matrix with *m* objects and *n* variables, *T* is the score matrix with dimensions (*m* × *f*), *P^T^* is a transposed matrix of loadings with dimensions (*f* × *n*), and *E* is a matrix of the residual variance (*m* × *n*) not explained by the first *f* principal components. On the whole, the first few principal components (PCs) frequently sufficiently describe data variance and reveal the groups of objects.

Hierarchical clustering analysis (HCA) allows for the assessment of (dis)similarities among objects within the variable space. Therefore, it is essential to determine the similarity metric and the method of linking resulting sub-clusters beforehand. The results are displayed as a dendrogram, where the *X*-axis represents the indices of the clustered objects and the *Y*-axis reflects the linkage distances between the connected objects. Additionally, this visualization technique can be utilized on empirical data arranged according to the sequence of objects, creating color-coded variable maps. A combined interpretation of objects arranged using the Ward linkage method alongside the color-coded variable maps aids in evaluating the (dis)similarity of objects based on the input parameters.

### 3.6. Similarity-Based Activity Landscape Index

The quantitative assessment of the similarity-related structure–activity landscape index (SALI) can be represented by the following equation:

(2) $${SALI}_{x,y}=\frac{\left|A_{x}-A_{y}\right|}{1-sim(x,y)}$$

where $A_{x}$ and $A_{y}$ are the activity profiles for the x-th and y-th molecule and *sim*(*x,y*) is the pairwise similarity measure. The Tanimoto coefficient was utilized for estimating similarity based on fingerprints, with the structural pairwise molecular relatedness calculated as follows:

(3) $$T\left(x,y\right)=\frac{n_{xy}}{\left(n_{x}+n_{y}-n_{xy}\right)}$$

where $n_{xy}$ is the number of bits set into 1 shared in the fingerprint of molecule *x* and *y*, $n_{x}$ is the number of bits set into 1 in molecule *x*, and $n_{y}$ is the number of bits set into 1 in molecule *y*, respectively.

### 3.7. Molecular Docking

The crystal structure of human acetylcholinesterase (AChE) in complex with tacrine, resolved at 2.85 Å, was downloaded from the PDB repository (PDB code: 7XN1). Apart from crystal water, all heteroatoms, including the 1,2,3,4-tetrahydro-9-acridinamine (tacrine, THA) molecule, were removed from the binding site of chain A, prior to docking with the AutoDock Vina 1.2.0 program (Scripps Research, San Diego, CA, USA). Using PyMol (Schrödinger, Inc., New York, NY, USA) and Chimera 1.19 software (RBVI, San Francisco, CA, USA), the ligands and enzyme structures were first prepared and corrected in a pdbqt file format, incorporating the calculated Gasteiger charges. A grid box measuring 15 × 15 × 15 Å was centered on the nitrogen atom of the THA amine group. Docking simulations in AutoDock Vina generated different poses (by default nine) from a single conformer (an energy-optimized molecule). The resulting molecular conformations and orientations (poses), including the preferred torsion angles and rotatable bonds, were subsequently assessed using the united-atom (UA) scoring function. To visualize the anticipated 2D and 3D binding modes, Schrödinger Maestro graphical tools (Schrödinger, Inc., New York, NY, USA) and the Protein-Ligand Interaction Profiler (PLIP) were utilized, respectively.

## 4. Conclusions

The study evaluated the inhibitory activity of ten quinobenzothiazinium salt derivatives against AChE and BChE enzymes. All the tested compounds showed significant inhibition of AChE with IC_50_ values in the range of 0.03–0.658 µM. The most effective derivative was 5,8,10-trimethyl-12*H*-quinolino[3,4-*b*][1,4]benzothiazin-5-ium chloride (**3d**), which surpasses the clinically used rivastigmine and galantamine in its properties and achieves comparable activity to tacrine and donepezil. For BChE, IC_50_ values ranged between 0.34 and 4.25 µM, with 5,9-dimethyl-12*H*-quinolino[3,4-*b*][1,4]benzothiazin-5-ium chloride (**3b**) showing the highest activity. In general, it can be stated that a number of compounds are more effective in inhibiting BChE than rivastigmine and galantamine. Selectivity indices confirm a clear preference for AChE inhibition. Furthermore, cytotoxicity assays on HFF-1 cells confirmed the safety of these active compounds. In silico pharmacokinetic profiling showed that all derivatives adhere to Lipinski’s rule of five, suggesting high gastrointestinal absorption. With the exception of derivatives **3g** and **3h**, all compounds are predicted to effectively cross the blood–brain barrier.

A similarity evaluation of structural properties and AChE inhibitory response values for the analyzed molecules **3a**–**3j** was reported. The activity profiles were analyzed through a similarity-mediated approach using the PCA and HCA procedures on a collection of Dragon descriptors. A visual representation of chemical similarity alongside biological activity was created based on the systematic profiling of SALI. The identification of activity cliffs is heavily reliant on the availability of chemically similar compounds that exhibit significant variations in activity. Thus, AChE values were incorporated into the SALI computation. In fact, the distance-based similarity assessment confirmed the existence of three distinct clusters of compounds, characterized by low (molecules **3e**–**3i**), moderate (molecule **3d**), and high (molecule **3j**) Euclidean distances. Moreover, the most potent molecule **3d** with double –CH_3_ groups in position 8 and 10 of the benzene ring can potentially form activity cliffs with inactive molecule **3i** that have one ethyl substituent attached to the heterocyclic nitrogen.

Target-oriented molecular docking was utilized to rearrange the spatial distribution of the investigated compounds within the binding pocket of the AChE. Although the marketed drug tacrine and the molecules **3a**–**3j** exhibit structural differences, special emphasis was put on the most potent AChE inhibitor **3d**, due to its high binding affinity towards AChE (comparable with tacrine). It revealed three dominant types of interacting modes: hydrophobic, hydrogen-bonding and π-stacking ones. An automated docking procedure positioned energetically favorable pose 1 of tacrine in a way that promoted hydrophobic and π-stacking interactions, with tacrine and molecule **3d** sandwiched between Trp86 and Tyr337. Noticeably, the docking protocol anticipated multiple hydrophobic interactions for the most active molecule **3d**, involving the following amino acid residues: Trp86, Tyr133, Tyr337, Trp439, Tyr449, and Ile451. The amine group of tacrine and the non-aromatic nitrogen of compound **3d** appear essential as hydrogen bond donors, facilitating the formation of a hydrogen bond with the carbonyl oxygen of His447. In practice, the water molecules in the binding cavity were preserved, leading to the formation of a bridged hydrogen bond between the ring nitrogen of tacrine, crystal water 709, and Ser125. Still, this bonding pattern was not observed for the most active molecule **3d**.

## Figures and Tables

**Table 1:** List of investigated compounds and their A/BChE inhibition (IC_50_ [µM]), selectivity to AChE (SI) and selectivity to BChE (SI*), and cytotoxicity IC_50_ [μM] on HFF-1 cells.

No.	Structure	AChE IC_50_ [µM]	BChE IC_50_ [µM]	SI/SI*	HFF-1 IC_50_ [µM]
**3a**	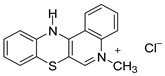	0.288 ± 0.005	0.522 ± 0.013	1.8/0.55	42.6 ± 1.8
**3b**	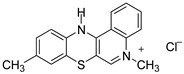	0.115 ± 0.006	0.343 ± 0.012	3.0/0.34	35.8 ± 1.2
**3c**	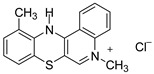	0.239 ± 0.014	2.695 ± 0.042	11.3/0.09	>100
**3d**	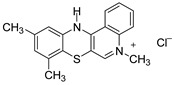	0.030 ± 0.001	1.989 ± 0.047	66.3/0.02	>100
**3e**	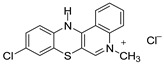	0.083 ± 0.002	1.292 ± 0.017	15.6/0.06	>100
**3f**	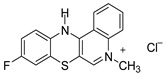	0.116 ± 0.009	0.668 ± 0.009	5.8/0.17	>100
**3g**	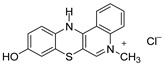	0.167 ± 0.006	4.254 ± 0.042	25.5/0.04	>100
**3h**	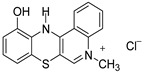	0.147 ± 0.008	0.781 ± 0.007	5.3/0.19	>100
**3i**	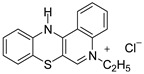	0.658 ± 0.036	1.696 ± 0.015	2.6/0.39	30.8 ± 1.6
**3j**	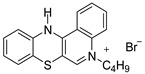	0.264 ± 0.011	1.020 ± 0.019	3.9/0.26	>100
**rivastigmine**		56.10 ± 1.41	38.40 ± 1.97		
**galantamine**		1.54 ± 0.02	2.77 ± 0.15		
**tacrine**		0.038 ± 0.002	0.004 ± 0.00006		
**donepezil**		0.015 ± 0.0001	0.0314 ± 0.001		
**cisplatin**					20.6 ± 0.8

SI—ratio IC_50_(BChE)/IC_50_(AChE); SI*—ratio IC_50_(AChE)/IC_50_(BChE).

**Table 2:** Physicochemical properties, Lipinski’s rule of five parameters, gastrointestinal absorption, and bioavailability of investigated compounds and standards.

Comp.	MW [g/mol]	Clog*P*	HBD	HBA	Violation	tPSA [Å^2^]	GI Absorption	Bioavailability Score
**3a**	300.81	1.71	1	0	0	41.21	high	0.55
**3b**	314.83	2.29	1	0	0	41.21	high	0.55
**3c**	314.83	2.16	1	0	0	41.21	high	0.55
**3d**	328.86	2.70	1	0	0	41.21	high	0.55
**3e**	335.25	2.55	1	0	0	41.21	high	0.55
**3f**	318.80	2.45	1	1	0	41.21	high	0.55
**3g**	316.81	1.53	2	1	0	61.44	high	0.55
**3h**	316.81	1.77	2	1	0	61.44	high	0.55
**3i**	359.28	2.18	1	0	0	41.21	high	0.55
**3j**	387.34	2.78	1	0	0	41.21	high	0.55
**rivastigmine**	250.34	2.34	0	3	0	32.78	high	0.55
**galantamine**	287.35	1.92	1	4	0	41.93	high	0.55
**tacrine**	198.26	2.59	1	1	0	38.91	high	0.55
**donepezil**	379.49	4.00	0	4	0	38.77	high	0.55

MW—molecular weight; Clog*P*—consensus log*P*_o/w_ (the arithmetic mean of the values predicted by the five proposed methods—XLOGP3, WLOGP, MLOGP, SILICOS-IT, and iLOGP); tPSA—topological polar surface area; HBA—number of H-bond acceptors; HBD—number of H-bond donors; GI—gastrointestinal.

## Data Availability

The original contributions presented in this study are included in the article/Supplementary Materials. Further inquiries can be directed to the corresponding authors.

## Supplementary Materials

The following supporting information can be downloaded at: https://www.mdpi.com/article/10.3390/molecules31081346/s1, Table S1. Binding affinities of pose 1 for the investigated compounds and standards; Figure S1. ^1^H NMR spectrum of 5,8,10-trimethyl-12(*H*)-quino[3,4-*b*][1,4]benzothiazinium chloride (**3d**) in DMSO; Figure S2. ^13^C NMR spectrum of 5,8,10-trimethyl-12(*H*)-quino[3,4-*b*][1,4]benzothiazinium chloride (**3d**) in DMSO; Figure S3. HRMS spectrum of 5,8,10-trimethyl-12(*H*)-quino[3,4-*b*][1,4]benzothiazinium chloride (**3d**).

## Abbreviations

The following abbreviations are used in this manuscript:

AChE	Acetylcholinesterase
AD	Alzheimer’s Disease
ADME	Absorption Distribution Metabolism Excretion
ACh	Acetylcholine
ATCh	Acetylthiocholine
BBB	Blood–Brain Barrier
BChE	Butyrylcholinesterase
BTCh	Butyrylthiocholine
CAMD	Computer-Assisted Molecular Design
ChEIs	Cholinesterase Inhibitors
CS	Chemical Space
DMSO	Dimethyl Sulfoxide
DTNB	5,5′-Dithiobis(2-Nitrobenzoic Acid)
GIT	Gastrointestinal Tract
HBA	H-Bond Acceptor
HBD	H-Bond Donor
HCA	Hierarchical Clustering Analysis
HFF-1	Human Dermal Fibroblasts
HRMS	High-Resolution Mass Spectrometry
IC	Inhibitory Concentration
K_M_	Michaelis Constant
log *P*	Logarithm of n-Octanol–Water Partition Coefficient
mD-QSAR	Multidimensional Quantitative Structure–Activity Relationship
mDS	Multidimensional Space
NMR	Nuclear Magnetic Resonance
PBS	Phosphate-Buffered Saline
PCA	Principal Component Analysis
PCs	Principal Components
PDB	Protein Data Bank
PLIP	Protein-Ligand Interaction Profiler
QSAR	Quantitative Structure–Activity Relationship
SALI	Structure–Activity Landscape Index
SAR	Structure–Activity Relationship
SBDD	Structure-Based Drug Design
SI	Selectivity Index
Tc	Tanimoto Coefficient
THA	Tacrine
tPSA	Topological Polar Surface Area
Vm	Maximum Velocity
