# Supplementary material for: Cholinesterase Inhibitory Activity of Alkylated Quinobenzothiazinium Salts
Source: Molecules. 2026 Apr 19;31(8):1346. doi: 10.3390/molecules31081346 (PMC13118828; doi:10.3390/molecules31081346)
Supplement: Supplementary file 1 [file molecules-31-01346-s001.zip › molecules-4264601-supplementary.pdf]

Supplementary materials

Cholinesterase inhibitory activity of alkylated quinobenzothiazinium salts

Sarka Stepankova [1,*], Andrzej Bak [2], Malgorzata Latocha [3], Violetta Kozik [2], Agata Kawulok [2,4], Josef Jampilek [2,5], and Andrzej Zieba [6,*]

1 Department of Biological and Biochemical Sciences, Faculty of Chemical Technology, University of Pardubice, Studentska 573, 532 10 Pardubice, Czech Republic
2 Institute of Chemistry, University of Silesia, Szkolna 9, 40-007 Katowice, Poland; andrzej.bak@us.edu.pl (A.B.), violetta.kozik@us.edu.pl (V.K.), agata.kawulok@us.edu.pl (A.K.), josef.jampilek@gmail.com (J.J.)
3 Department of Cell Biology, Faculty of Pharmaceutical Sciences in Sosnowiec Medical University of Silesia, Jedności 9, 41-200 Sosnowiec, Poland; mlatocha@sum.edu.pl (M.L.)
4 Department of Bone Marrow Transplantation and Oncohematology, Maria Sklodowska-Curie National Research Institute of Oncology, Gliwice Branch, Wybrzeze Armii Krajowej 15, Gliwice 44-101, Poland
5 Department of Chemical Biology, Faculty of Science, Palacky University Olomouc, Slechtitelu 27, 779 00 Olomouc, Czech Republic
6 Department of Organic Chemistry, Faculty of Pharmaceutical Sciences in Sosnowiec Medical University of Silesia, Jagiellońska 4, 41-200 Sosnowiec, Poland
* Correspondence: sarka.stepankova@upce.cz (S.S.); zieba@sum.edu.pl (A.Z.)

Table S1. Binding affinities of pose 1 for the investigated compounds and standards.

Comp.	Binding affinity of pose 1 [kcal/mol]
**3a**	-7.476
**3b**	-6.891
**3c**	-6.432
**3d**	-4.057
**3e**	-6.780
**3f**	-7.466
**3g**	-7.871
**3h**	-7.409
**3i**	-7.550
**3j**	-7.842
**rivastigmine**	-7.552
**galantamine**	-6.837
**tacrine**	-9.395
**donepezil**	-8.868

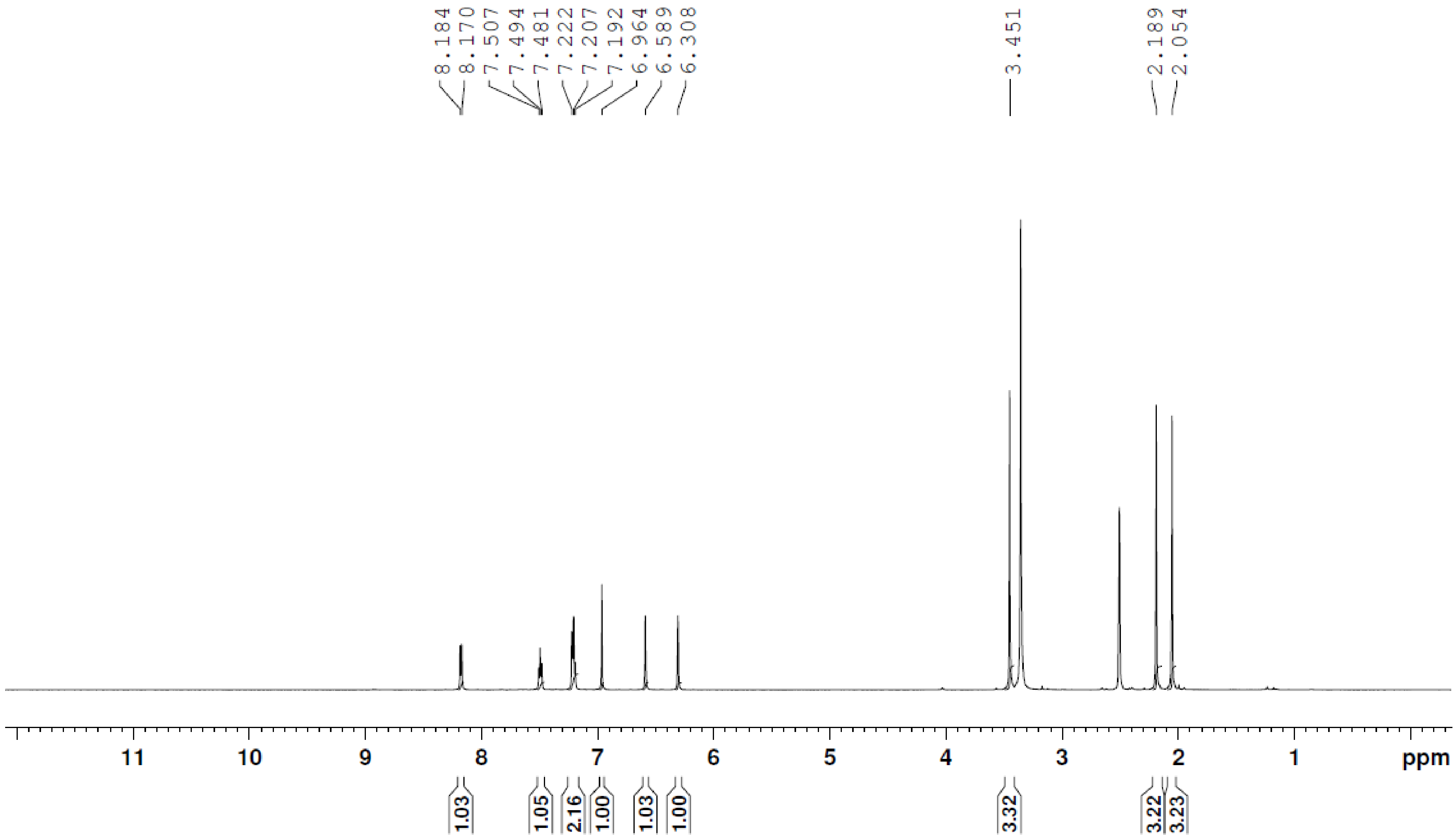

Figure S1. ^{1}H NMR Spectrum of 5,8,10-trimethyl-12(*H*)-quino[3,4-*b*][1,4]benzothiazinium chloride (**3d**) in DMSO.

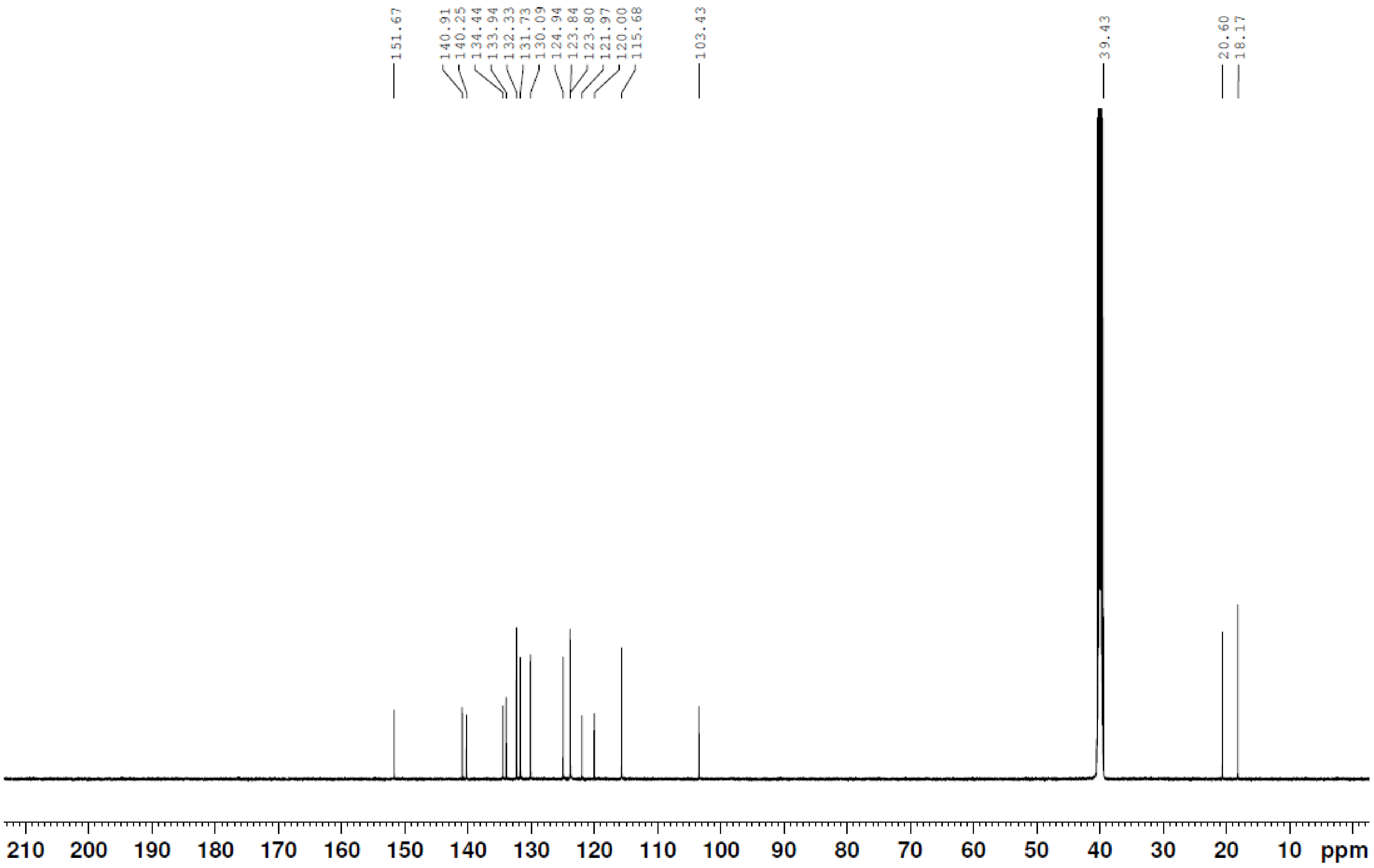

Figure S2. ^{13}C NMR Spectrum of 5,8,10-trimethyl-12(*H*)-quino[3,4-*b*][1,4]benzothiazinium chloride (**3d**) in DMSO.

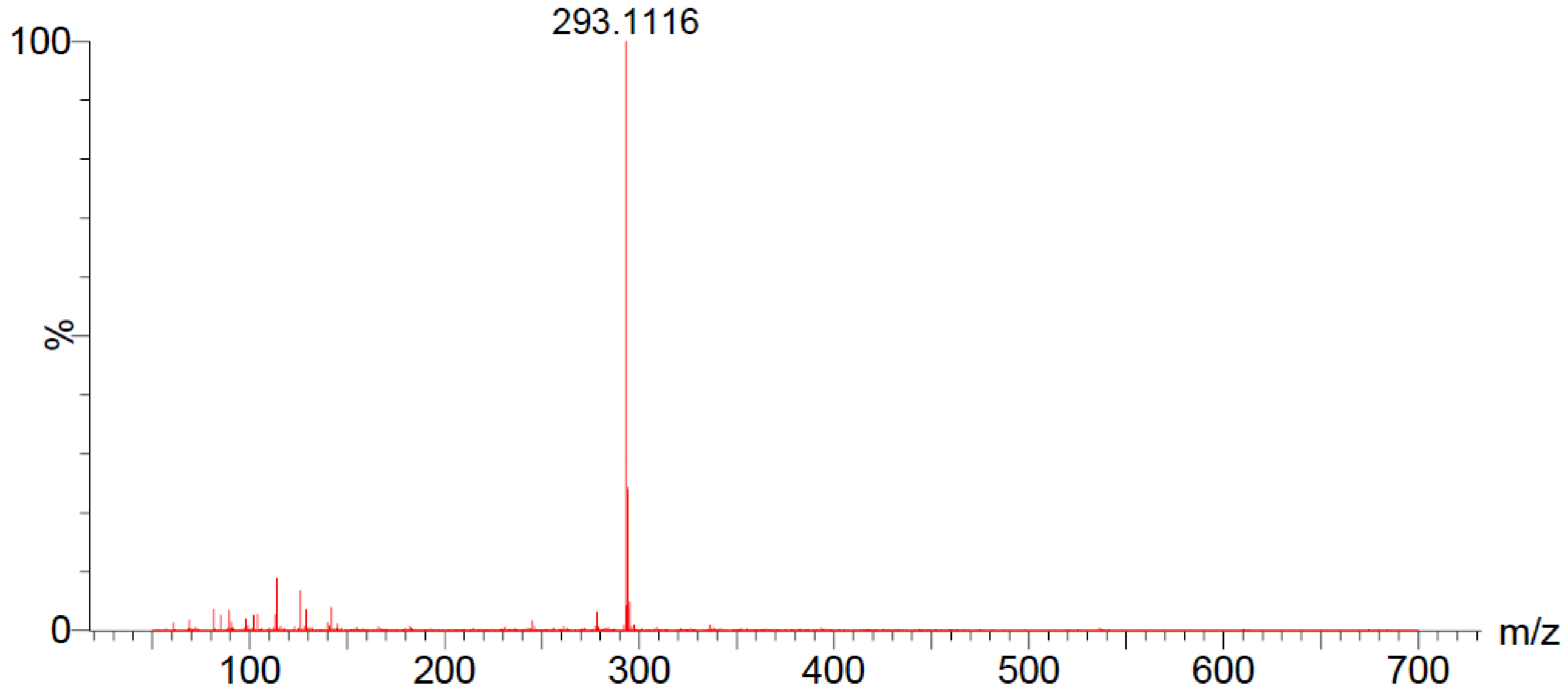

Figure S3. HRMS Spectrum of 5,8,10-trimethyl-12(*H)*-quino[3,4-*b*][1,4]benzothiazinium chloride (**3d**).
